# Staurosporine as an Antifungal Agent

**DOI:** 10.3390/ijms26199683

**Published:** 2025-10-04

**Authors:** Filipa C. Santos, Joaquim T. Marquês, Eva N. Santos, Rodrigo F. M. de Almeida

**Affiliations:** Centro de Química Estrutural, Institute of Molecular Sciences, Departamento de Química e Bioquímica, Faculdade de Ciências, Universidade de Lisboa, 1749-016 Lisboa, Portugal; fpsantos@ciencias.ulisboa.pt (F.C.S.); jmtmarques@ciencias.ulisboa.pt (J.T.M.); fc62847@alunos.ciencias.ulisboa.pt (E.N.S.)

**Keywords:** antimycotic activity, indolocarbazole, mode of action, pathogenic fungi, drug-resistant fungal infections, protein kinase inhibitors, fungal plasma membrane, fluorescent alkaloid, sphingolipid-enriched domains (SLEDs)

## Abstract

Staurosporine (STS) was discovered in 1977 by Omura and colleagues during a chemical screening for microbial alkaloids. It was the first indolocarbazole compound isolated from a soil-dwelling bacterium, *Streptomyces staurosporeus*. STS was also found to have antifungal activity, but its potent protein kinase (PK) inhibitory properties, perhaps the most extensively characterized biochemical feature of STS, were only revealed nearly a decade after its discovery. Thereafter, STS has been studied mainly for its anticancer potential with foreseen applications ranging from biomedical (e.g., antiparasitic) to agricultural (e.g., insecticidal). Interestingly, the recent discovery that STS induces apoptosis in the filamentous fungus *Neurospora crassa* renewed interest in this molecule as a scaffold for antifungal drug development. Studies in fungi and mammalian cell lines suggest that, in addition to PK inhibition, other modes of action are possible for STS. These may involve the targeting of membrane lipid domains and/or alterations of membrane biophysical properties. Here, the studies on the action of STS and its natural and synthetic derivatives against diverse fungal species, since its discovery to the present day, are critically reviewed and discussed with the aim of highlighting their advantages, limitations to be overcome, conceivable mechanisms of action, and potential as antifungal chemotherapeutic agents.

## 1. Staurosporine: An Indolo[2,3-a]carbazole Alkaloid with Anticancer and Antifungal Activity

Antifungal drug resistance is a significant societal concern, with mortality rates associated with resistant fungal infections increasing rapidly, particularly in hospital environments and among immunocompromised patients [1,2,3,4,5]. Each year, 6.5 million cases of invasive fungal infections result in nearly 3.8 million deaths, of which approximately 2.5 million are directly attributable to fungal pathogens [6]. This information is based on data from more than 85 countries in which ca. 90% of the world’s population live [7]. Drug-resistant strains—especially multidrug-resistant *Candida auris*—are notable contributors, with mortality rates of 29–62%, with the highest rates observed in Intensive Care Unit settings among immunocompromised patients [8,9]. Understanding the mechanisms of action underlining the antifungal activity of chemotherapeutic agents available and developing new antifungal therapies is, therefore, crucial.

Actinomycetes are a highly valuable source of antibiotics and other biologically active compounds with significant commercial importance, including vitamins, alkaloids, plant growth regulators, enzymes, and enzyme inhibitors [10,11]. These Actinobacteria are known for producing antibiotics belonging to various chemical classes and exhibiting diverse biological activities [12]. Among them, *Streptomyces* spp. stands out as the most prolific producers of antibiotics. In fact, around 60% of the antibiotics discovered during the 1990s, as well as most antibiotics used in agriculture, were derived from *Streptomyces* [11,13].

Staurosporine (STS) was originally isolated from the fermentation broth of *Streptomyces staurosporeus* (strain AM-2282) [14]. Since then, STS has been isolated from several other microorganisms, such as *Streptomyces roseoflavus* [15], as will be described further ahead in this review. Renowned for its diverse bioactivities, STS exhibits potent antifungal [14,16] and antitumoral [17] properties. The genus *Streptomyces* comprises spore-forming, filamentous, Gram-positive bacteria within the phylum Actinobacteria [18]. It represents one of the most ubiquitous bacterial genera in diverse environments, distinguished by its remarkable capacity to biosynthesize a wide array of natural products with substantial biological activities through their secondary metabolism [19]. These metabolites hold significant relevance in medicine, environmental applications, food industries, and agricultural practices [20,21].

**Figure 1:**
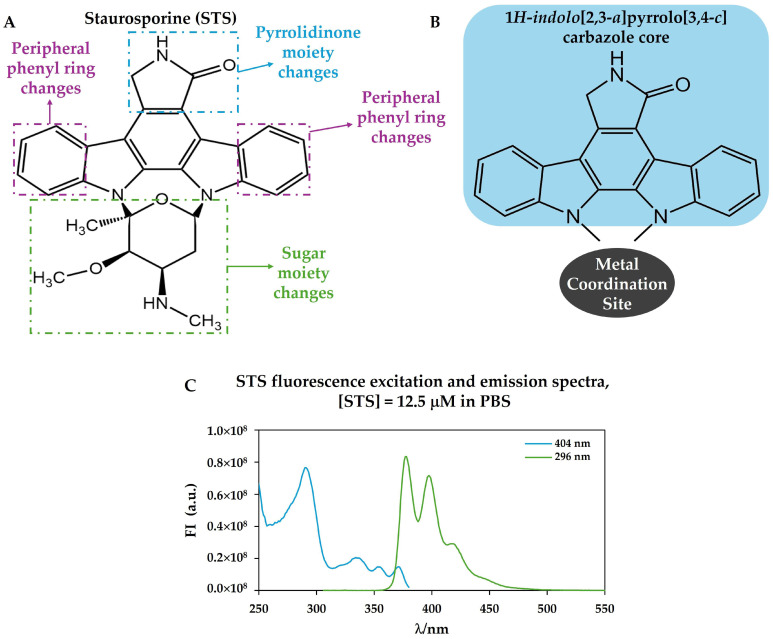
(**A**) Chemical structure of staurosporine, STS, (AM-2282) with the identification of the possible chemical modifications found in natural compounds structurally related to STS and used to derive synthetic analogues; (**B**) structure of 1*H*-indolo[2,3-a]pyrrolo[3,4-c]carbazole core (highlighted with blue shading) showing the typical site for metal complex formation; and (**C**) STS fluorescence excitation spectrum (blue) with emission at l = 404 nm and STS fluorescence emission spectrum (green) with excitation at l = 296 nm, in phosphate-buffered saline (PBS).

The prototypical alkaloid STS belongs to the indolocarbazole family, a diverse class of natural compounds identified in a wide range of organisms, including actinomycetes, cyanobacteria, fungi, slime moulds, and marine invertebrates [22], which is characterized by a fusion of indole and carbazole rings (Figure 1A). Notably, only the indolo[2,3-a]carbazole isomer is found in nature [23]. This structural motif is a key component of numerous natural products, many of which exhibit significant biological activities, although their specific biological functions remain largely unexplored [24]. The core structure of STS and its analogues can be defined by the indolo[2,3-a]pyrrolo[3,4-c]carbazole (Figure 1A,B) [23].

**Figure 2:**
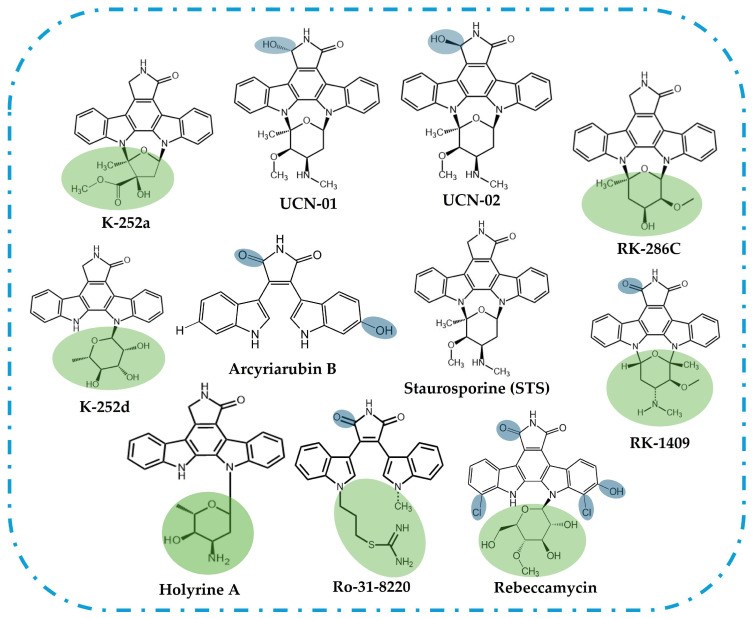
Representation of the chemical structures of “open” bisindolylmaleimide and “closed” indolo[2,3-a]carbazole analogues of STS [43,44,45,46,47]. The shaded areas highlight the structural differences in relation to STS, in the sugar residue/groups attached to nitrogen in the indolocarbazole moiety (green) or other modifications (blue).

Structurally, these molecules are distinguished by a core framework that occurs as either an “open” bisindolylmaleimide (e.g., arcyriarubin B, Ro-31-8220) or a “closed” indolo[2,3-a]carbazole (e.g., tjipanazole F2, rebeccamycin, and STS) (Figure 2) and to which metals can coordinate (Figure 1B) [22,25,26]. These alkaloids have attracted significant scientific interest due to their remarkable structural diversity and a broad spectrum of biological activities, such as antitumor, neuroprotective, antibacterial, antifungal, antiviral, and hypotensive [27].

In the first work reporting the isolation of STS, it was recognized that it is very active against yeast and multicellular fungi in vitro, but only weakly active against bacteria [14]. This discovery expanded the number of classes of alkaloids with potential applications against fungal infections [28]. Regarding human pathogenic fungi, STS showed the lowest minimal inhibitory concentration (MIC) value against *Candida pseudotropicalis* and *Aspergillus brevipus* (3.13 μg/mL in both cases). Regarding *Candida albicans* and *Aspergillus niger*, the MIC values were higher, being 6.25 μg/mL and 25 μg/mL, respectively [14]. This subject will be further discussed in Section 5, where a compilation of MIC values is presented. STS antifungal activity is attributed to its capacity to disrupt essential cellular processes including cell membrane integrity and stress response pathways [29,30,31,32].

STS is widely recognized as one of the most potent protein kinase (PK) inhibitors, with an in vitro half-maximal inhibitory concentration (IC_50_) in the nanomolar range [33]. PKs play a central role in signal transduction, mediating both extracellular and intracellular signalling pathways [34]. Moreover, they regulate all aspects of the cell cycle through the phosphorylation of critical proteins. By inhibiting PKs, which play pivotal roles in fungal signalling networks, STS interferes with fungal growth and proliferation [33,35,36]. Additionally, STS is a well-known inducer of programmed cell death (PCD) across various systems, including neuronal cells (e.g., [37]), protozoans (e.g., [38]), human macrophages (e.g., [39]), and the filamentous fungus *Neurospora crassa* [31,40,41,42].

Nevertheless, in *C. albicans*, for example, STS unexpectedly induces filamentation by activating the Cyr1-cAMP-PKA signalling cascade, suggesting that STS may trigger alternative pathways not involving PK inhibition [35]. Furthermore, it was found that *N. crassa* plasma membrane (PM) contains sphingolipid-enriched domains (SLEDs) [48], a specific type of ergosterol-depleted PM domain first found in the yeast *Saccharomyces cerevisiae*, where lipids are tightly packed in a very rigid gel phase [49,50], and that they are involved in the response to STS [29]. Possible alternative mechanisms of action for STS will be discussed in Section 6.

In this review, we aim to explore the potential of STS as an antifungal agent and as a scaffold for novel antifungal compounds by bringing together information on the biological activities related to its antifungal action and delving into its mode of action. Understanding the biochemical mechanisms by which STS can stop fungal growth or cause fungal cell death may pave the way for the development of novel therapeutic strategies to address the growing challenge of antifungal drug resistance.

## 2. Brief History of Staurosporine: From Discovery to Biological Activity Disclosure

In 1977, during investigations into bioactive compounds of microbial origin and the ongoing exploration of alkaloid production by actinomycetes, Omura et al. achieved the first isolation of STS by solvent extraction and silica gel chromatography [14] from soil gathered at the Japanese city of Mizusawa.

The compound was initially designated AM-2282 after the producing strain *Streptomyces* sp. AM-2282. Since then, this microorganism has undergone multiple taxonomic reclassifications. It was renamed *Streptomyces staurosporeus* AM-2282 in 1977, *Saccharothrix aerocolonigenes* subsp. *staurosporea* AM-2282 (NRRL 11184, ATCC 55006) in 1995, and *Lentzea albida* in 2002 [51]. STS received its current name in 1978 following the elucidation of its relative stereochemistry through the X-ray crystallographic analysis of its methanol solvate structure [52]. However, its absolute configuration was clarified only sixteen years later by Funato et al. [53].

The activity of STS was initially tested against eight bacterial species, including *Staphylococcus aureus*, *Bacillus subtilis*, and *Escherichia coli*, as well as twelve fungal species, such as *C. albicans*, *Aspergillus fumigatus*, and *Cryptococcus neoformans*. Notably, the latter three fungal species—against which STS demonstrated activity—are now listed in the 2025 WHO fungal priority pathogens list, which aims to guide research, development, and public health action [5]. STS showed potent activity against *C. albicans* and moderate activity in the case of *A. fumigatus* and *C. neoformans*. Additionally, our collaborators from *Oporto* showed that the combination of rotenone and STS is effective against *N. crassa* as well as against the common pathogens *A. fumigatus* and *C. albicans*, which points to its importance as an antifungal agent [31].

The shift towards oncobiology may be linked to a 1986 report describing STS as “a potent inhibitor of Ca^2+^/phospholipid-dependent protein kinase (PKC)” [33]. This study also suggested that STS probably inhibits PKC by direct binding [33]. More recent studies have further elucidated its inhibitory mechanism, revealing that STS acts as a potent competitive inhibitor by strongly binding to the ATP-binding pocket of almost all kinases in their active conformation [54,55,56]. Since the mid-1980s, PKs have served as the primary cellular targets for the development of anticancer agents [57]. For a comprehensive review on the antitumor effects of STS and other indolocarbazoles, the reader is referred to the following references: [58,59]

It was quickly recognized that STS acts as a nonspecific inhibitor of PKs, as it also inhibits cAMP-dependent protein kinases (PKAs), cGMP-dependent protein kinases (PKGs), and tyrosine PKs at similar concentrations [60,61]. STS is thus a pan-kinase inhibitor, affecting over 253 kinases with a dissociation constant below 3 μM, including those present in blood plasma [14,33,62,63,64]. This lack of selectivity is a major cause of the well-documented toxicity of STS, namely through its ability to induce apoptosis in a large variety of mammalian cell lines, which significantly hinders the molecule’s drugability.

The 2000s marked a period of significant exploration into the antifungal properties of STS, with numerous studies focusing on its mechanisms of action. In 2000, a group of researchers found several loci mutations that affect the sensitivity of the yeast *S. cerevisiae* to STS [65]. Yoshida and colleagues demonstrated that STS sensitivity is closely linked to ergosterol and glycolipid biosynthesis pathways, as well as vacuolar functionality [51]. The analysis of mutations influencing the sensitivity of *S. cerevisiae* to STS has provided significant insights into its mode of action. Researchers have identified that STS sensitivity is closely related to v-ATPase function—a yeast enzyme that plays an important role in pH homeostasis. Mutants defective in the v-ATPase assembly exhibited STS sensitivity, indicating that proper vacuolar function is necessary for the export of STS from the cytosol into the vacuole. In addition, they found that mutations in vacuolar protein sorting and vacuolar membrane ATPase genes, along with genes encoding ABC transporters, impact drug sensitivity. They suggested that STS is exported from the cytosol by several ABC transporters and/or H^+^/drug antiport (which relies on the proton gradient established by the v-ATPase) [65].

In 2006, Park et al. investigated the antimicrobial activity of STS using a growth inhibition assay on microtiter plates [16]. This work reported the isolation of STS from *Streptomyces roseoflavus* and established its in vitro and in vivo anti-oomycete activity against *Phytophthora capsici,* a pathogenic oomycete that causes devastating diseases in a wide range of plant hosts. STS demonstrated the complete inhibition of mycelial growth in the plant pathogenic fungi *P. capsici*, *Rhizoctonia solani*, and *Corynespora cucumerinum*, with MIC values ranging from 1 to 50 μg/mL [51].

Substantial efforts to develop STS analogues with improved selectivity were initiated and continue to this day. Furthermore, the development of these analogues is essential to address the compound’s significant toxicity, as STS has been shown to exhibit cytotoxic effects even at very low concentrations. As mentioned, STS is a highly potent yet non-selective PK inhibitor that has been widely studied, but it has no approved therapeutic applications in humans due to its broad activity and associated toxicity. Among its derivatives, the best known clinically approved agent is Midostaurin (PKC412, RYDAPT^®^), a semisynthetic benzoyl STS, approved in the United States and Europe for use in combination with standard chemotherapy in adults with newly diagnosed acute myeloid leukemia harbouring *FLT3* mutations and as a monotherapy for aggressive systemic mastocytosis, systemic mastocytosis with an associated hematologic neoplasm, and mast cell leukemia [66]. Other STS analogues, such as UCN-01 (7-hydroxystaurosporine—Figure 2) and lestaurtinib (CEP-701), have been evaluated in early-phase clinical trials for various cancers, including solid tumours and acute myeloid leukemia, but to date, they have not achieved regulatory approval [67,68].

## 3. Biological Sources and Natural and Synthetic Analogues of Staurosporine

As mentioned at the beginning of this review, STS is a secondary metabolite originally isolated from the fermentation broth of *S. staurosporeus* (AM-2282) [14] and later obtained from other *Streptomyces* spp. [28], which are renowned for their ability to synthesize several bioactive natural products.

A compound named LS-A24 was obtained from the Actinomycete strain LS-A24 with physiological and biochemical features resembling those of *S. roseoflavus*. This compound presented a chemical structure identical to AM-2282 (STS) (Figure 1A).

LS-A24 showed a high level of antifungal activity against various plant pathogenic fungi and oomycete pathogens. This compound was tested against several microorganisms (e.g., *Fusarium oxysporum* f.sp. *lycopersici*, MIC = 50 μg/mL; *S. cerevisiae*, MIC = 1 μg/mL; and for *C. albicans*, the growth was not inhibited at a concentration of 100 μg/mL) [16].

Additionally, a wide range of STS analogues have been isolated from cultures of various microorganisms. These analogues exhibit structural diversity, with modifications occurring in the sugar moiety, peripheral phenyl rings, pyrrolidinone ring, or through a combination of alterations in these structural components (Figure 1A and Figure 2) [23].

UCN-01 (7-hydroxystaurosporine) is an example of a bioactive STS analogue that was isolated from *Streptomyces* sp. N-126, a strain that also produces its stereoisomer UCN-02 (7-epi-hydroxystaurosporine) [46]. UCN-01 is a PK inhibitor (e.g., PKC and PKA) (Table 1) that presents antifungal activity against *C. albicans* and *C. neoformans*. Similarly to STS, UCN-01 was shown to be synergistic with fluconazole, a characteristic that seems to be related to its structural similarities with STS [69]. However, the UCN-02 stereoisomer showed less PK inhibitor capacity compared to UCN-01, and, despite the lack of data regarding its antifungal activity, it will likely be a less active antifungal agent.

K-252a, previously named K-252, is a metabolite isolated from the culture broth of *Nocardiopsis* sp. K-252. K-252a, like STS, allows the growth of *Streptomyces griseus* even at high concentrations but inhibits its aerial mycelium formation and pigmentation. It was shown that both STS and K-252a inhibit the phosphorylation of several proteins (e.g., PKC, Table 1) [73].

K-252d is structurally related to K-252a (3′-(*S*)-epi-K-252a) and was already isolated from the culture broth of *Nocardiopsis* sp. K290 [71] and *Streptomyces* sp. ZS-A121 [74]. It was shown that it also inhibits PKC, however, with smaller effects than K-252a [71]. Furthermore, K-252d exhibited activity against *C. albicans* [74]. Holyrine A was isolated from *Streptomyces* sp. ZS-A121 and, alongside K-252d, also exhibited activity against *C. albicans* [74].

RK-286c, also known as 4′-demethylamino-4′-hydroxystaurosporine, Figure 2, is a weak inhibitor of PKC (Table 1). It was isolated from the bacterium *Streptomyces* sp. RK-286 [72]. Additionally, it exhibits a weak antifungal activity against *Piricularia oryzae* [28]. Structurally, RK-286c is a derivative of STS, differing by the presence of a hydroxyl group and the absence of a methylamino group at specific positions.

## 4. STS Biosynthetic Pathway

**Figure 3:**
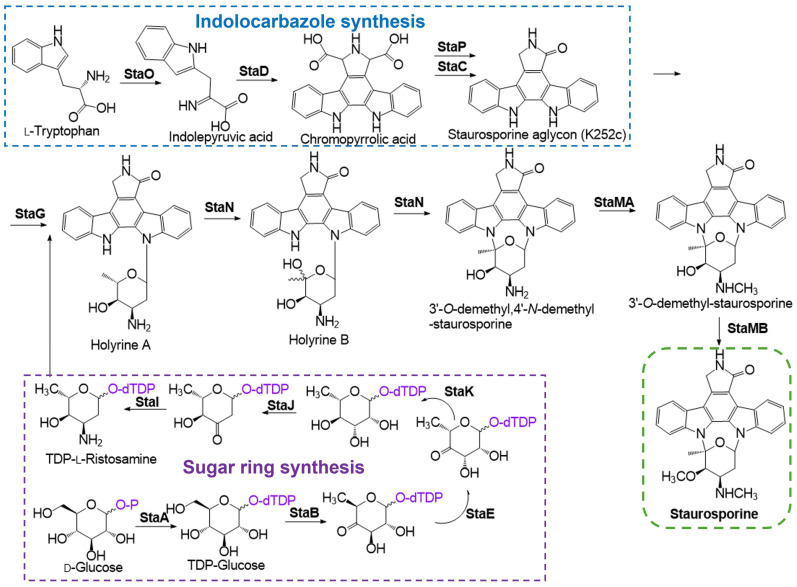
Schematic representation of STS biosynthetic pathway, with the representation of indolocarbazole synthesis with l-Tryptophan as primary substrate and sugar ring synthesis with d-Glucose as primary substrate (adapted from [24,51]). The enzyme StaO, l-amino acid oxidase, initiates the catalyzing process through the conversion of l-tryptophan into the imine form of indole-3-pyruvic acid (IPA imine). Subsequently, StaD, a chromopyrrolic acid (CPA) synthase facilitates the coupling of two IPA imines to produce chromopyrrolic acid. The formation of the indolocarbazole core, a key structural feature of STS, is mediated by StaP (CYP245A1), which transforms chromopyrrolic acid into three indolocarbazole compounds: STS aglycone (K-252c), 7-hydroxy-252c, and arcyriaflavin A. This transformation occurs via intramolecular C-C bond formation and oxidative decarboxylation. Structural studies of the P450 enzyme StaP reveal that its heme group removes two electrons from the indole ring, generating an indole radical. This radical undergoes intramolecular coupling to form the C-C bond, establishing the indolocarbazole core [75]. The presence of StaC, primarily directs the formation of K-252c. Subsequent modifications involve StaG, coding for a *N*-glycosyltransferase, which catalyzes the formation of an *N*-glycosidic bond between N-13 and C-6′, followed by the action of StaN, coding for a cytochrome P450 oxygenase that facilitates an additional C-N bond between N-12 and C-2′. Together, these enzymes convert K-252c into 3′-*O*-demethyl,4′-*N*-demethyl-STS through the intermediates holyrine A and B. Finally, StaMA, an *N*-methyltransferase, and StaMB, a 4-*O*-methyltransferase, complete the STS biosynthesis by methylating the compound. StaMA catalyzes *N*-methylation of 3′-*O*-demethyl,4′-*N*-demethyl-staurosporine, while StaMB performs *O*-methylation, resulting in the formation of the final product, STS [24,51].

The biosynthesis of STS is governed by a complex enzymatic pathway involving indolocarbazole precursors, which originate from l-Tryptophan and d-Glucose as primary substrates (Figure 3).

In the STS biosynthetic pathway (Figure 3), a complex enzymatic system is involved: StaO (l-amino acid oxidase, catalizes the conversion of of l-Tryptophan to IPA imine [76]), StaD (CPA synthase, involved in oxidative modifications essential for the formation of the characteristic aglycone structure of STS [77]), StaP (CYP245A1, transforms CPA into three indolocarbazoles, being STS aglycone one of them, which is a precursor in the pathway [76]), StaC (contributes to the core scaffold tailoring of STS [51]), StaA (d-glucose-1-phosphate thymidyltransferase [76]), StaB (dTDP-glucose-4,6-dehydratase [76]), StaE (3,5-epimerase, catalyzes the epimerization at specific positions of sugar intermediates, crucial for configuring the sugar moiety attached to the STS aglycone [78]), StaK (4-ketoreductase, reduces 4-keto groups in sugar intermediates during the biosynthesis of the deoxy sugar component of STS [78]), StaJ (2,3-dehydratase, removes water molecules from sugar intermediates, forming a keto sugar, facilitatingthe insertion of an amino group in the sugar moiety [78]), StaI (3-aminotransferase, transfers amino groups to sugar intermediates, introducing amino functionalities essential for the biological activity of STS [78]), StaG (*N*-glycosyltransferase, that catalyzes the formation of the *N*-glycosidic bond [76]), StaN (a cytochrome P450 oxygenase responsible for C-N bond formation between aglycone and deoxysugar at the C-5′, contributing to the formation of the final product [77]), StaMA (*N*-methyltransferase, methylates nitrogen atoms within the sugar moiety, contributing to the final structural configuration of STS [78]), and StaMB (4-*O*-methyltransferase, methylates oxygen atoms at the 4′-position of sugar intermediates, further modifying the sugar moiety to achieve the complete structure of STS [78]).

## 5. Antifungal Activity of Staurosporine and Related Compounds

The antimicrobial screening undertaken by a conventional agar dilution method in the first study describing the identification of STS by Omura et al. revealed that this indolocarbazole has a broad spectrum of antifungal activity [14]. Since then, the antifungal activity of STS and its natural and synthetic analogues have been determined against a variety of fungal species, including human pathogens [74,79,80,81,82] and phytopathogenic fungi [15,16,83,84,85].

STS analogue K-252a exhibited potent inhibitory activity on PKC, with an IC_50_ of 32.9 nM. K-252a also inhibited calmodulin-activated enzymes from bovine brain and heart, although the effect was weaker than the one on PKC, with an IC_50_ of 97.5 μM [43].

In a 1991 study [86], researchers isolated and characterized STS-sensitive and temperature-sensitive mutants of *S. cerevisiae*, leading to the identification of a gene, *STT1*, which was found to be critical for bud formation and cellular growth. Further analysis established that *STT1* is identical to *PKC1*. STS was shown to inhibit yeast cell growth by targeting the *STT1* gene product, arresting cell cycle at the G2/M phase, which suggests that it functions as the *S. cerevisiae* homologue of PKC. Furthermore, a 1994 study demonstrated that *S. cerevisiae PKC1* encodes a PKC homologue with a substrate specificity similar to that of mammalian PKC, underscoring the challenges in achieving kinase selectivity [87]. Interestingly, in mammalian cells, STS induces cell cycle arrest at both the G1 and G2 phases [88]. Additionally, STS seems to inhibit several cellular functions in yeast, as in this study, mutations in at least ten distinct genes have been shown to result in an STS-sensitive phenotype [86].

### 5.1. Antifungal Activity of Staurosporine Against Phytopathogens

Table 2 summarizes MIC values found in the literature for STS and related compounds. It was reported that STS has antifungal activity (Table 2) against several microorganisms, such as *C. albicans* (MIC = 6.25 μg/mL), *A. niger* (MIC = 25 μg/mL), *A. fumigatus* (MIC = 12.5 μg/mL), and *C. neoformans* (MIC = 50 μg/mL) [14]. Nonetheless, considering the large amount of research articles involving STS, only a scarce number of works have addressed the antifungal properties of STS and its naturally occurring analogues or synthetic derivatives. In some of these works, the activity of STS or of its analogues highlights their potential as antifungal chemotherapeutic agents, deserving further investigation and investment. Interestingly, STS isolated from the LS-A24 strain was very effective in inhibiting the Phytophthora disease on pepper plants at 500 μg/mL, a concentration at which the compound does not show any phytotoxicity in this plant [16]. In the same study, the activity of STS against different fungi was evaluated, and the data obtained revealed a strong growth inhibition of several microorganisms, including *P. capsici* (MIC = 1 μg/mL), *B. subtilis* ssp. *subtilis* (MIC = 10 μg/mL), and *Xanthomonas vesicatoria* (MIC = 50 μg/mL) (Table 2) [16]. In line with its ability to inhibit the Phytophthora disease on pepper plants, STS was shown to be responsible for the antifungal activity against *Fusarium oxysporum* f.sp. *cucumerinum*, the fungal pathogen responsible for *Fusarium* vascular wilt of cucumbers, in a study where the antimicrobial potency of Actinobacteria against *Fusarium oxysporum* f.sp. *cucumerinum* was evaluated [83]. Still regarding its fungicidal activity against phytopathogens, STS was shown to inhibit the mycelium development of the deadly phytopathogen *Magnaporthe oryzae* Triticum, a wheat blast fungus [15]. STS significantly reduced conidia production by *Magnaporthe oryzae Triticum*, and at 300 μg/mL, a total absence of conidiophores was observed [15]. Moreover, STS proved to be a strong inhibitor of conidial germination [15]. In another study, STS and some structurally related carbazoles, including 7-oxo-staurosporine, isolated from marine *Streptomyces* spp., impaired the motility of *Plasmopara viticola* zoospores in a dose- and time-dependent manner [84]. STS at 0.05 µM (0.02 μg/mL) and 7-oxo-staurosporine at 0.4 µM (0.19 μg/mL) promoted the complete inhibition of zoospore motility [84]. At 0.05 µM (0.02 μg/mL), STS fully inhibited zoosporogenesis, and at 2 µM (0.93 μg/mL), it completely suppressed *P. viticola* sporulation [84]. It was observed that the mycelial growth of *Pleurotus ostreatus*, a common edible mushroom, was completely inhibited at 9.6 µM (4.48 μg/mL) of STS [85]. At 0.54–0.96 µM (0.25–0.45 μg/mL) periodic constrictions, whose frequency were dependent on the STS concentration, occurred in the mycelia [85]. Swellings of hyphal tips and of subapical regions, although less frequent, were also observed due to the interaction with STS. Moreover, above 6.5 µM (3.03 μg/mL), STS fully blocked the regeneration of protoplasts [85].

Plants are susceptible to various biotic stresses induced by bacteria, viruses, fungi, parasites, harmful insects, and weeds. From the examples explored above detailing the antiphytopathogenic effects of STS and analogues, their relevance to develop more resilient agricultural practices becomes evident. Considering that the largest percentage of crop loss is attributed to insects, the insecticidal activity of STS against lepidoptera [89], a major phytophagous insect group, also represents another important biological activity of STS that encourages its use in agriculture.

### 5.2. Antifungal Activity of Staurosporine Against Human Pathogens

Besides its antifungal activity against several phytopathogens, STS also presents a strong activity against human fungal pathogens, either alone or through synergistic interactions with other antifungals.

In a study where it was shown that *Streptomyces* sp. BV410 produces STS, it was confirmed that this compound presents potent antifungal activity against various species of *Candida*, *C. albicans*, *C. krusei*, *C. parapsilosis*, and *C. glabrata* with MICs of 0.098, 0.39, 0.098, and 0.024 μg/mL (Table 2), respectively [80]. The MIC value of STS against *C. albicans* found by these authors is considerably lower than that initially reported by Omura et al. [14], which may be related to the methodology used to evaluate the antifungal activity. While Mojicevic et al. assessed antifungal activity by standard disc diffusion assays [80], Omura et al. employed a conventional agar dilution method [14]. In another study where the antimicrobial assay was also performed by the microdilution method, STS exhibited a MIC of 50 μg/mL against *C. albicans* [74]. Holyrine A (streptomholyrine A), a recently discovered STS analogue from marine-derived Actinomycete *Streptomyces* sp. ZS-A121 exhibits stronger antifungal activity against *C. albicans* than STS (MIC of 12.5 vs. 50 μg/mL) (Table 2) [74]. The antifungal activity against *C. albicans* of another three analogues of STS was investigated in the same study and, among them, K-252d also displayed a more potent antifungal activity than STS (MIC = 25 μg/mL) (Table 2) [74]. Another study has also highlighted that 7-oxo-staurosporine, another STS analogue, exhibited selective growth inhibitory activity against the mycelial form of *C. albicans* in a dose-dependent manner (MIC = 25 μg/mL) [79], despite its weak antifungal activity against *Magnaporthe grisea* [81]. In addition, 7-oxo-staurosporine has also shown strong antifungal activity against the mycelial form of various species of *Candida*, namely *C. krusei*, *C. tropicalis*, *C. lusitaniae*, with MICs of 3.1, 50 and 12.5 μg/mL, respectively (Table 2) [79]. Interestingly, 7-oxo-staurosporine did not induce any growth inhibition of the yeast forms of these organisms up to 200 μg/mL. Thus, 7-oxo-staurosporine seems to be a selective inhibitor of the mycelial form of *Candida* spp. A promising result presented in that study is that, despite its strong anticandidal activity, 7-oxo-staurosporine is not toxic to SPR-ICR mice up to 60 mg/kg [79]. In a study where the antimicrobial activities of twenty-two substances structurally related to STS were examined against *Streptomyces chartreusis*, *S. griseus*, *Bacillus cereus*, *E. coli*, *C. albicans,* and *B. cinerea*, only the two chloro-indolocarbazole compounds were active against *C. albicans* [73]. Interestingly, their fungicidal activity was comparable to that observed with chloro-indolocarbazole tjipanazoles, which are compounds isolated from the blue-green alga *Tolypothrix tjipanasensism* bearing structural resemblances with STS [82].

STS also exhibits an important synergistic interaction with antifungals, including drugs in current clinical use. In a drug screen study to identify molecules that abolish azole resistance of both an *S. cerevisiae*-resistant mutant and a *C. albicans* clinical isolate, STS stood out as one of the 7 hits among the 1280 pharmacologically active compounds tested [36]. Moreover, another two of the seven hits were inhibitors of PKC. Upon the deletion of *PKC1* in *C. albicans*, fungistatic drugs became fungicidal; furthermore, reduced virulence in a mouse model was also observed [36]. In the same study, the authors observed that pharmacological or genetic impairment of *PKC1* result in hypersensitivity to multiple drugs that target the synthesis of ergosterol (the major sterol in the PM of fungi) including azoles, morpholines, and allylamines. Indeed, STS enhanced the efficacy of antifungals targeting the synthesis of ergosterol, namely, fluconazole (which inhibits Erg11p), fenpropimorph (which inhibits Erg2p and Erg24p) and terbinafine (which inhibits Erg1p) and targeting the cell wall, namely micafungin. While in *S. cerevisiae*, the pharmacological inhibition of PKC signalling blocked the activation of a key regulator of membrane stress responses, calcineurin, in *C. albicans*, *PKC1* and calcineurin independently regulate resistance via a common target [36]. In line with the set of results explored above, the addition of STS to wild-type *C. glabrata* cells in a medium containing fluconazole led to a decrease in recoverable viable cells from 90 to 15% [90]. STS also interacts synergistically with caspofungin in the growth inhibition of pathogenic *Aspergillus* clinical isolates [91]. Caspofungin is an antifungal agent that interferes with glucan synthesis and cell wall formation [91]. The ability to act synergistically with fluconazole was also observed for the STS analogue UCN-01, though this compound did not exhibit the same synergistic effect with caspofungin [69].

Besides allowing it to bypass drug resistance in *C. albicans*, STS also influences its morphogenesis by inducing filamentation in the absence of any other signal [35]. STS induced filamentous growth of wild-type *C. albicans* under conditions that are distinct from those that usually induce filamentation, such as a rich medium at 30 °C vs. carbon-limiting Spider medium or serum, which requires a concurrent increase in temperature to 37 °C [35]. Similarly to the described above for *Pleurotus ostreatus* [85], STS-induced filaments exhibited constrictions, in contrast to the hyphae generated in the Spider medium or serum [35]. The authors thus concluded that STS induces filaments with characteristics that implicate distinct cellular routes from that engaged by other filament-inducing conditions [35], including those that induce filamentation at 30 °C, such as geldanamycin [92]. In fact, STS is the first cue reported to be able to induce filamentous growth in a strain lacking *PKC1*, since the deletion of *PKC1* did not abrogate STS-induced filamentation as it does for all the other stimuli tested by the authors, including the RPMI medium at 37 °C, Spider medium at 37 °C, 10% serum at 37 °C, and geldanamycin at 30 °C [93]. Moreover, the authors observed a defect in septin-ring formation, thus concluding that cell cycle kinases involved in cell division are potential STS targets in STS-induced filamentation [35]. It is known that the morphological transition from the yeast cell to hyphae is one of the virulence factors involved in the pathogenesis of *C. albicans* [94].

The metabolite K-252a has been shown to inhibit the formation or function of compounds involved in sporulation, possibly through interactions that lead to their inactivation. These effects are associated with the inhibition of protein phosphorylation, highlighting a potential regulatory link between phosphorylation events and both aerial mycelium and pigment formation in *S. griseus* [73].

Several questions thus arise concerning the full understanding of the impact of the data described. Is the yeast to hyphae morphogenic change induced by STS in *C. albicans* also related to virulence, similarly to the morphological transition induced by the serum? And is the morphological transition induced by STS accompanied by an increase in ergosterol levels, as observed in the case of *N. crassa*? [95,96]. Our findings in Santos et al. [29] suggest that STS prevents mycelia formation or at least delays the increase in ergosterol levels that accompanies this developmental stage, hence the question is whether, in this case, hyphae are also formed via a different route and the composition of the membrane is less rich in ergosterol or not? And what other changes could there be in the cell membrane that make it more or less susceptible to other antifungal drugs, namely polyenes that form pores in ergosterol-rich membranes? If so, and considering the mode of action of polyene antibiotics, does STS promote an increase in the sensitivity to polyenes under conditions where it promotes the transition from yeast to mycelium? These are open questions and the ability to answer them will allow to build a more complete understanding of the mechanisms underlining STS antifungal action.

### 5.3. Strategies to Improve Activity and Overcome Toxicity

To overcome the issue of STS toxicity, several strategies have been or may be implemented. Drug delivery systems that provide specific targeting and improve drugs’ pharmacodynamics and pharmacokinetics can be developed, ultimately reducing the required dose to be effective and overcoming toxicity issues [64]. Similar results may be achieved with combination therapies, considering the synergistic effects mentioned in Section 5.2, allowing for a stronger therapeutic efficacy at lower doses, reducing toxicity, and decreasing resistance to other antifungal drugs. Another strategy is to perform co-treatment with molecules that reduce toxicity or alleviate side effects specifically caused by STS. For example, it has been shown that curcumin reduces STS cytotoxicity against rat hippocampal neurons in primary cultures by decreasing the production of reactive oxygen species (ROS) and increasing the levels of antiapoptotic factors induced by STS while decreasing the levels of proapoptotic factors [97]. Curcumin also has a protective effect in retinal ganglion cells, being able to prevent STS-induced cell death at certain curcumin-to-STS ratios in vitro and in vivo [98]. Interestingly, curcumin, widely known for its antioxidant and anti-inflammatory properties, also possesses significant antifungal activity. Its fungicidal activity has been shown against a variety of both phytopathogens and clinical isolates of *Candida* species [99]. Reports in the literature suggest that the mechanisms behind curcumin antifungal activity may involve intracellular acidification via the inhibition of H^+^-efflux [100] or inhibition of ergosterol biosynthesis [101]. Curiously, and similarly to the cases described for STS in Section 5.2, curcumin also displays synergistic effects with antifungal agents in clinical use [99]. However, to the best of our knowledge, studies involving the combination of curcumin and STS have not been conducted.

An important property of STS is that it affects chemoresistance to other drugs. In Section 5.2, examples with antifungal agents were presented. This feature is more general as, for example, in colon, breast, and ovarian cancer cell lines, the overexpression of resistance-related factors promoted by treatment with cisplatin (the benchmark and most widely used first-line anticancer chemotherapeutic agent) [102] seems to be counteracted by STS. The experiments were carried out under cisplatin challenge conditions that led to absent or marginal proliferation inhibition. This recapitulates a situation that can happen in a patient undergoing therapy, where some cancer cells may be subjected to sub-optimal doses of the drug. Moreover, cell proliferation assays indicated that STS and cisplatin have additive effects or that STS was able to render the cells more susceptible to cisplatin. The approaches outlined with these examples allow for the use of STS or its derivatives’ dosages with less serious side effects. In summary, the STS scaffold can be further explored for co-treatments, either to prevent indolocarbazole unwanted toxicity or to act additively/synergistically with other drugs.

The most important strategy is perhaps the design of analogues that retain or even increase the intended bioactivity while reducing toxicity, in particular, derivatives or analogues that are much more specific on their action, reducing the range of molecular targets (mostly protein kinases). The antifungal properties of STS and analogues/derivatives remain largely unexplored and unexploited. This gains special relevance given the current ability to synthesize a myriad of STS derivatives. In fact, the studies discussed above highlight that using STS as a scaffold and introducing (minor) structural changes can significantly improve the compound antifungal activity. In particular, it is fit to mention that, in 2020, Gayler and co-workers developed a method that enables the preparation of STS derivatives functionalized at C2 and C10 of the indolocarbazole aromatic region [103], which opens a new route to explore novel STS derivatives with potent antifungal activity.

## 6. Antifungal Modes of Action

To develop improved antifungal therapies, it is imperative to fully disclose the mode by which currently known compounds with established antifungal activity operate. In the case of STS, as stated throughout this review, its potent PK inhibitory properties lay at the foundation of several of its known fungistatic or fungicidal activities. However, as also referred to, this lack of specificity poses several challenges, as it can trigger different and/or multiple responses which depend on the specific experimental conditions and can also lead to cytotoxic effects against human cells.

### 6.1. Protein Kinase Inhibition and Induction of Apoptosis

To better understand these effects, it is important to consider how PKs function at the molecular level. PKs are broadly classified into two categories: tyrosine kinases and serine–threonine kinases [23]. Tyrosine kinases catalyze the phosphorylation of the phenolic group of tyrosine residues, while serine–threonine kinases target the hydroxyl groups of serine and threonine residues. Despite their differences in substrate specificity, all PKs utilize ATP as the phosphorylating agent, requiring distinct regions within their active sites for ATP binding and substrate recognition [23].

In fungi and mammals, PK-based signalling pathways are evolutionarily conserved and play crucial roles in regulating processes such as stress adaptation, drug resistance, and pathogenesis [35,87]. Among them, serine/threonine PKs are particularly noteworthy for their essential role in cell wall remodelling during fungal growth [87]. These kinases share homology with the α, β, and γ isoforms of mammalian PKC and localize to sites of polarized growth, including the mother–daughter bud neck [87]. Notably, the loss of *PKC1* function leads to a severe cell lysis defect caused by impaired cell wall construction, underscoring its critical role in maintaining fungal cell integrity and viability [87].

Exposure to STS induces cell death in the filamentous fungus *N. crassa* by triggering a rise in cytosolic Ca^2+^ levels [104,105]. However, in response to STS, *N. crassa* upregulates the expression of the ABC transporter ABC-3, which localizes at the PM and actively pumps STS out of the cell [41], possibly the most important drug-resistance mechanism of this fungus in response to STS. Indeed, a mutant strain lacking this ABC transporter (*abc3*) is highly sensitive to STS. In the same study, significant changes were observed in the *m*RNA levels encoding for several enzymes involved in lipid metabolism, as well as (signalling) proteins that interact with the membrane and may influence membrane domain formation and properties. As already mentioned, STS is a broad-spectrum PK inhibitor, and this function in mammalian cells induces apoptosis through both caspase-dependent and caspase-independent pathways [106]. In *N. crassa*, phospholipase C seems to be an essential player coordinating the mobilization of Ca^2+^ to the cytosol during STS-induced cell death, as cells lacking the phospholipase C gene *PLC*-2 show a higher survival rate and no STS-induced rise of cytosolic Ca^2+^ [104]. The importance of extracellular Ca^2+^ during STS-induced fungal PCD is reinforced by the observation that cell death in *N. crassa* is impaired in a Ca^2+^-free medium and inhibited by the excess of Ca^2+^ [30]. In agreement with these observations, a similar protection from fungal PCD was observed by the presence of an excessive amount of extracellular Ca^2+^ in occidiofungin-treated *S. cerevisiae* cells and chitosan-treated *N. crassa* cells [107,108]. The *N. crassa* mitochondrial respiratory chain is part of the intracellular Ca^2+^ dynamics regulation upon an STS challenge [105,109]. More specifically, the deletion of certain subunits of complex I of the respiratory chain, e.g., *NUO51* and *NUO14*, disrupts Ca^2+^ signalling after STS stimulus, resulting in hypersensitivity to STS [31]. The chemical disruption of other components of the mitochondrial respiratory chain also led to defective Ca^2+^ responses during PCD caused by STS [105].

In addition to the changes in Ca^2+^ levels, the export of glutathione (GSH) and the accumulation of ROS are crucial events during fungal cell death, e.g., ROS accumulation has been shown to occur after STS treatment [31]. Furthermore, *N. crassa* cells, during STS-induced PCD, export GSH, which seems to be one of the causes of cell death [42]. This leads to an imbalance in the intracellular GSH/glutathione disulfide ratio favouring ROS accumulation and the oxidation of cellular components. Notably, STS-induced cell death is avoided through supplementation with exogenous GSH or its precursor *N*-acetyl-cysteine [31]. Interestingly, the addition of exogenous GSH or *N*-acetyl-cysteine blocked the STS-induced intracellular Ca^2+^ response, indicating that it is dependent on ROS [105].

### 6.2. Alterations at the Plasma Membrane Level and in Fungal Developmental Processes

The biophysical properties of the PM during conidial germination were thoroughly characterized in *N. crassa* [48]. In this work, it was found that, in agreement with low ergosterol levels before the conidia/mycelium transition, the PM of *N. crassa* conidia is essentially devoid of sterol-rich liquid ordered domains but presents gel phase SLEDs. SLEDs are highly rigid domains that are rich in sphingolipids but essentially devoid of ergosterol; they are present at the fungal PM but absent in mammalian cells, rendering them of potential paramount importance in the fight against fungal infections [50]. The membrane biophysical properties in *N. crassa* conidia are highly dynamic, undergoing progressive changes in the course of germination, concomitant with new membrane biogenesis and lipidome alterations [48]. To understand any phenomena involving the conidial PM, it is essential to know a priori these properties and how they are modulated throughout the developmental stage; thus, this work paved the way to study in detail the involvement of membrane lipid domains and biophysical properties in response to STS in filamentous fungi. It was found that STS does not interact or, if so, it does very weakly with lipid bilayers [29] in a study that took advantage of STS’s intrinsic fluorescence, as shown in Figure 1C. However, it was also shown that STS has an impact on membrane lipid domains in *N. crassa* in a manner that depends on the conidial stage development and duration of the STS challenge [29]. As previously stated, in *N. crassa*, STS induces the regulated overexpression of the ABC transporter ABC-3, which is located at PM, and pumps STS out of the cell. To understand the role of PM biophysical properties in the fungal drug response, wild-type *N. crassa* and the mutant lacking the ABC transporter (*abc3*) were treated with STS during the early and late stages of conidial development. After 1 h of treatment with STS, there is an increase in the abundance of the highly ordered SLEDs, which leads to greater fluidity in other regions of the membrane. Significant changes in SLEDs were also observed after 15 min of incubation with STS but were essentially opposite to those observed for the 1 h treatment, suggesting different types of responses depending on the duration of exposure to the drug. Interestingly, the intracellular levels of STS are higher after 15 min than 1 h, probably due to the export through ABC-3 [41].

The effects of STS on membrane properties that are more dependent on ergosterol levels also depend on the stage of development [29]. No significant changes were observed in cells grown for 2 h, at which the PM is essentially devoid of ergosterol, in clear contrast to what happens during longer growth times. For the latter, the differences were more pronounced for the longer treatment with STS and rationalized considering that the drug prevents the increase in the ergosterol/glycerophospholipid ratio that normally occurs in the late conidial stage/transition to the mycelial stage. This can be perceived as a halt in the development induced by STS treatment after 5 h of growth, involving ergosterol, and pointing to a role for lipid rafts possibly related to the regulated overexpression of the ABC-3 transporter, which pertains to a protein family that has been typically associated with lipid rafts [110]. In summary, our results suggest the involvement of ordered membrane domains in the mechanisms of response to STS in *N. crassa*.

Regarding the relation of SLEDs with the response to STS in fungi, it is interesting to recall the previously mentioned work on the induction of filamentation in *C. albicans* by STS through a pathway independent of *PKC1* inhibition [35]. Such filamentation involved a defect on the septin-ring formation. Septins are essential for both cell membrane and cell wall remodelling in fungi. In yeast, septins bind exclusively to liquid ordered-phase domains [111], while in *Aspergillus nidulans*, a filamentous fungus, core septins form functional assemblies at sites of membrane and cell wall remodelling that are dependent on a normal sphingolipid metabolism [112]. On the other hand, it was shown that in *N. crassa* conidia, the fluidity of these disordered regions is also influenced by STS [29]. It was recently suggested that curvature contributes to the binding of septins, along with high fluidity of the liquid disordered regions, and that these regions of high curvature may be stabilized by the presence of rigid and very low diffusivity SLEDs [111]. Again, in *N. crassa*, STS changed SLEDs properties. Thus, the changes that STS is known to induce in filamentous fungi membrane biophysical properties may be related to the morphogenetic alterations involving septins.

## 7. Concluding Remarks and Future Research

The discovery of STS marked a significant milestone in drug development, introducing a new paradigm in the search for bioactive compounds from microbial sources—specifically the ‘Compound first—Bioactivity second’ approach pioneered by Omura’s group [59]. However, this also highlighted the complex reality that moving from an initial compound with promising biological activity to a clinically viable therapeutic agent takes time and requires multiple approaches. The therapeutic application of this compound is limited due to its lack of selectivity, which results in toxicity. Consequently, the focus has progressively shifted to the synthesis of STS derivatives that preserve the potent biological effects while enhancing selectivity and minimizing toxicity. Bisindolylmaleimide compounds, which are STS analogues derived from its aglycon moiety, are highly selective inhibitors of multiple PKC isoenzymes in human promyelocytic leukemia HL-60 cells [47], in opposition to the broad PK inhibition by STS. Interestingly, for one of these derivatives, Ro-31-8220 (Figure 2), potent apoptotic activity in HL-60 cells is not dependent on PKC inhibition, suggesting a different mechanism not only for this molecule but for STS itself [68].

K-252a, K-252b, K-252c, RK-286d, and RK-286c are microbial-derived STS analogues that exhibit lower, comparable, or even higher PKC inhibitory activity than STS. Nevertheless, unlike STS, they do not display antimicrobial properties. This observation suggests that the antimicrobial activity of STS may be independent of, or not exclusively attributable to, its protein kinase inhibitory capacity.

The antifungal potential of STS and its analogues is compelling but an underdeveloped field. With the global rise in fungal resistance and a stagnant antifungal pipeline, further research into kinase inhibitors, through rational design, combination therapy, and membrane-targeted delivery, could give rise to new therapeutic strategies. While traditionally classified as a kinase inhibitor, STS exhibits intriguing properties suggesting multiple modes of action, with consequences on both membrane and cell wall organization and integrity, particularly in fungi. Future research should explore the biophysical interactions of STS with fungal membrane models (e.g., large unilamellar vesicles and giant unilamellar vesicles composed by/or containing fungal lipids and proteins, such as septins). Membrane biophysical properties and alteration of the lipid and protein composition of pathogenic fungi PM in response to STS or its related compounds should also be pursued.

The fluorescence properties of STS analogues should also be explored—which STS derivatives are fluorescent? This is important because fluorescence can be used to trace the compound inside fungal structures and give important insights into its mechanisms of action.

The studies of STS on *N. crassa* conidia were pioneering in our understanding of the involvement of lipid domains in the response to this antifungal compound. However, further studies are required in order to fully disclose this involvement. Namely, it will be crucial to analyze the relative fractions of cells that are undergoing cell death and apoptosis under the STS challenge and to understand if the alteration observed in SLEDs and other membrane properties are a general part of the PCD pathway or are specific to the STS response. It will also be crucial to analyze the response of the *abc3* mutant strain, which is highly sensitive to STS due to its inability to efficiently export the drug. Finally, investigating the STS response in *N. crassa slime*, a cell wall-less strain, will give important contributions for distinguishing the role of the cell membrane and of the cell wall in the fungal response to the drug.

In yeast *S. cerevisiae*, the pH of the medium was shown to influence sensitivity to STS, indicating that environmental conditions play a role in STS’s effectiveness. Several mutations were found that lead to an increased sensitivity of *S. cerevisiae* cells to STS or different responses under different pH conditions. The researchers proposed that utilizing these mutations alongside adjustments to medium conditions could enable the identification of gene mutants with increased sensitivity to STS at lower concentrations [65,86]. Through this, it was emphasized that combining specific mutations and conditions could lead to the discovery of new compounds, such as those derived from STS, that target key proteins and create opportunities for drug development.

Finally, an important route to explore is the synthesis of STS analogues that are more lipophilic and thus might be able to interact more strongly with the fungal PM. Recently, this approach has been applied to ketoconazole, leading to a derivative that is not only able to inhibit the specific enzyme target of azoles but is also very efficient in permeabilizing the membrane [113,114], which conferred the activity against ketoconazole-resistant strains of *S. cerevisiae* and *C. albicans* to this compound [115].

## Figures and Tables

**Table 1:** STS biological sources and half-maximal inhibitory concentration of the listed protein kinases (PK).

Name	Biological Source	Protein Kinase	IC_50_ (nM)	References
Staurosporine (AM-2282)	*Streptomyces staurosporeus*	PKC PKA	2.7 8.2	[15,23,45]
UCN-01 (7-hydroxystaurosporine)	*Streptomyces* sp. N-126	PKC PKA	4.1 42	[45,46,69]
UCN-02 (7-epi-hydroxystaurosporine)		PKC PKA	62 250	[45,46,70]
K-252a	*Nocardiopsis* sp. K-252	PKC	32.9	[23]
K-252d	*Nocardiopsis* sp. K-290	PKC	337	[71]
RK-286c	*Streptomyces* sp. RK-286	PKC	3000	[28,72]

**Table 2:** STS and analogues’ biological sources and antifungal activity (minimal inhibitory concentration (MIC) values against the indicated fungal species).

Compound Name	Biological Source	Antifungal Activity	MIC (μg/mL)	Conventional Dilution Method	References
Staurosporine (AM-2282)	*Streptomyces staurosporeus*	*C. albicans*	6.25	Petri dish	[14]
		*C. pseudotropicalis*	3.13		
		*Saccharomyces sake*	3.13		
		*A. niger*	25		
		*A. brevipus*	3.13		
		*A. fumigatus*	12.5		
		*Trichophyton rubrum*	6.25		
		*Trichophyton mentagrophytes*	25		
		*C. neoformans*	50		
		*Sclerotinia cinerea*	0.78		
		*P. oryzae*	0.78		
		*Pleorotus ostreatus*	4.48	Petri dish	[85]
		*Protoplasts*	>3.03		
		*N. crassa*	~6	Petri dish	[31]
STS	*S. roseoflavus LS-A24*	*P. capsici*	1	24-well microtiter dish	[16]
		*R. solani*	1		
		*Colletotrichum orbiculare*	1		
		*Botrytis cinerea*	50		
		*Cladosporium cucumerinum*	1		
		*Didymella bryoniae*	10		
		*F. oxysporum* f.sp. *lycopersici*	50		
		*S. cerevisiae*	1		
		*Bacillus subtilis* ssp. *subtilis*	10		
		*X. vesicatoria*	50		
STS	*Streptomyces* sp. *B5136*	*Plasmopara viticola (zoospores)*	0.02	Petri dish	[84]
RK-1409 (7-oxostaurosporine)			0.19		
		*Candida krusei*	3.1		[79]
		*Candida tropicalis*	50		
		*Candida lusitaniae*	12.5		
STS	*Streptomyces* sp. *BV410*	*C. albicans*	0.098	Petri dish	[80]
		*C. krusei*	0.39		
		*Candida parapsilosis*	0.098		
		*Candida glabrata*	0.024		
Holyrine A	*Streptomyces* sp. *ZS-A121*	*C. albicans*	12.5	Petri dish	[74]
K-252d			25		

## Data Availability

No new data were created or analyzed in this study. Data sharing is not applicable to this article.

## Abbreviations

The following abbreviations are used in this manuscript:

GSH	Glutathione
PCD	Programmed Cell Death
PK	Protein Kinase
PM	Plasma Membrane
PKA	Protein Kinase A (cAMP-dependent protein kinase)
PKC	Protein Kinase C (Ca^2+^/phospholipid-dependent protein kinase)
PKG	Protein Kinase G (cGMP-dependent protein kinases)
ROS	Reactive Oxygen Species
SLEDs	Sphingolipid-Enriched Domains
STS	Staurosporine
IPA	indole-3-pyruvic acid
CPA	Chromopyrrolic acid
